# Antagonistic effects of lycopene on cadmium-induced hippocampal dysfunctions in autophagy, calcium homeostatis and redox

**DOI:** 10.18632/oncotarget.18249

**Published:** 2017-05-29

**Authors:** Fenghua Zhang, Suping Xing, Zongpeng Li

**Affiliations:** ^1^ Department of Operating Room, Linyi People's Hospital, Linyi 276000, Shandong, China; ^2^ Department of Oncology, Linyi People's Hospital, Linyi 276000, Shandong, China; ^3^ Department of Central Sterile Supply, Linyi People's Hospital, Linyi 276000, Shandong, China

**Keywords:** cadmium, lycopene, autophagy, ion-ATPases, redox

## Abstract

Cadmium (Cd), a widely existed environmental contaminant, was shown to trigger neurotoxicity by regulating autophagy, ion homeostasis and redox. Lycopene (LYC) is a natural substance with potent antioxidant capacity. Nevertheless, little is known about i) the relationship of Cd-induced neurotoxicity and autophagy, ion homeostasis as well as redox in the hippocampus; ii) the role of LYC in the regulation of hippocampal autophagy, ionic balance and antioxidant capacity during Cd exposure. Therefore, this study sought to investigate the Cd exposure-induced hippocampal dysfunctions for neurotoxicity, and the preventive potential of LYC on the hippocampus impairment by reversing the dysfunctions during the exposure. *In vivo* study with mice model demonstrated that Cd exposure increased gene expression of a wide spectrum of autophagy-related gene (ATG) and gene regulating autophagy in hippocampus. This suggests the activation of hippocampal autophagy mediated by Cd. Cd exposure also decreased Ca^2+^-ATPase activity, thus increasing intracellular Ca^2+^ concentration in hippocampus, indicating the possibility that Cd-induced autophagy requires the Ca^2+^ signaling. Moreover, Cd exposure triggered redox stress in hippocampus cells, as antioxidant enzyme activities were decreased while oxidative productions were promoted. Cd exposure led to severe cytotoxicity in hippocampus cells. Of important note, all the hippocampal dysfunctions upon Cd exposure were reversed by LYC treatment to normal situations, and exposure-induced neurotoxicity was abrogated. The *in vivo* findings were recapitulated relevantly in the mouse hippocampal neuronal cell line, TH22. In all, the above data imply that LYC could be a potent therapeutic agent in treating Cd-triggered hippocampal dysfunctions and subsequent cell damage.

## INTRODUCTION

Cadmium (Cd) is recognized as an important environmental contaminant. It exists in environment through battery, circuit board, plastics and fertilizers. Cd is widely dispersed in ecosystems worldwide and is transferred via food chains. As an extremely toxic mental, Cd can cause severe toxicity in most organs, leading to organ-derived diseases. One clinical study showed that environmental Cd exposure was associated with hepatic necro-inflammation, non-alcoholic fatty liver disease (NAFLD), and non-alcoholic steato hepatitis (NASH) in men, and hepatic necro-inflammation in women [1]. Cd potentiates diabetes-induced effects on kidney, and diabetic patients are more susceptible to renal tubular damage from low level of Cd expsorue than non-diabetic patients are [2]. Cd can also cause dysfunction of the central nervous system (CNS) after crossing the blood-brain barrier, leading to many neurodegenerative diseases [3]. More importantly, the neurotoxic effects may play a role in the systemic toxic effects of the Cd exposure, particularly the long-term exposure [4]. Hippocampus pathogenicity is a crucial factor contributing to most of the neurodegenerative diseases [5]. Moreover, previous reports demonstrated the impact of Cd on triggering hippocampal neurotoxicity [6, 7].

Lycopene (LYC), a nonprovitamin A carotenoid, is the most prevalent carotenoid in diet [8]. It is well known that LYC is a potent antioxidant. It is almost 100 times more efficient in quenching singlet oxygen than vitamin E. The asymmetric carbon skeleton and unsaturated bonds grants LYC with antioxidant capacity [9]. Another study indicates that the antioxidant activity of LYC is mainly dependent on its O^2−^ and -OH scavenging properties [10]. Studies have suggested that intake of LYC-enriched food results in many health benefits, such as decreased risks of cardiovascular diseases [11], nonalcoholic fatty liver disease [12], malignant brain tumor, brain injury, and other disease [13–15]. The promising effect of LYC on reducing these disease risks is mainly due to the potential of LYC on activating antioxidant enzyme activities and decreasing oxidant productions.

Autophagy is characterized by the conversion of LC3I to LC3II [16, 17]. It is required to maintain cellular homeostasis by performing “self-cleaning” catabolic process, which degrades waste protein aggregates and damaged organelles. Autophagy is associated with the excessive accumulation of reactive oxygen species (ROS) in dysfunctional mitochondria [18]. Mitochondrial derivation of ROS may activate autophagyby upregulating the necessary autophagy-related genes (ATGs) such as ATG4 to increase the formation of autophagosomes [19]. ATGs encode proteins that are required for macro-autophagy. There is growing evidence that the regulated expression of ATGs is linked to toxicity [20–22]. It was reported that Akt knockdown or inactivation with small molecule inhibitors increased autophagy [23], however, overexpression of Akt1 in macrophages potentiates autophagy [24]. MAPK pathway was a potential autophagy regulation pathway [25]. Furthermore, study showed that autophagy occurred through regulation of PRKAA signaling in endothelial cells, since the deletion of PRKAA can inhibit the autophagy [26].

The Ca^2+^ homeostasis is essential for the regulation of cellular bioprocess, such as uptake of nutrients, mitochondrial functions, cell differentiation and metabolism. The flux of intracellular Ca^2+^ was demonstrated to be mediated by the activity of Ca^2+^-ATPase [27]. Further study indicated that Ca^2+^-ATPase is able to replenish endoplasmic reticulum. Ca^2+^ stores during and after the action of agonists, which induce Ca^2+^ release [28]. Cd-induced toxicity requires the disruption of intracellular Ca^2+^ homeostasis [3]. However, the intracellular Ca^2+^ homeostatic change in Cd-exposed hippocampus is not known. In this study, we studied the Cd exposure-induced dysfunctional changes in hippocampus by investigating autophagy activation, Ca^2+^ flux and redox impairment; we also studied the potential of LYC on abrogating or attenuating Cd exposure-triggered hippocampal dysfunctions and neurotoxicity.

## RESULTS

### LYC abrogates cadmium exposure-induced hippocampal autophagy activation

**Figure 1:**
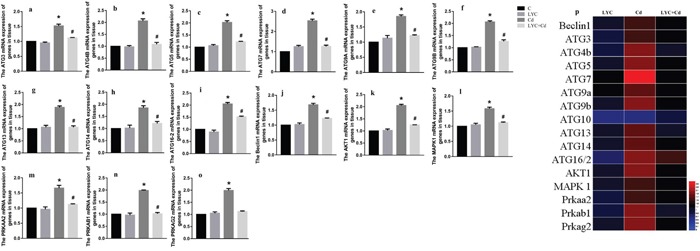
LYC abrogates cadmium exposure-induced hippocampal autophagy activation **(a-i)** ATG mRNAs, **(j)** beclin1, **(k)** Akt1, **(l)** MAPK1, **(m)** PRKAA2, **(n)** PRKAB1, **(o)** PRKAG2, **(p)** heatmap analysis. Bars represent mean ± S.D. (n=3). * represent LYC and Cd group compared to C group (P < 0.05); # represent LYC+Cd group compared to Cd group (P < 0.05).

Since Cd induces autophagy in mice brain [29], and induces autophagy by up-regulating autophagy-related gene (ATG) [3], we sought to know if cadmium activates autophagy in hippocampus by modulating ATG expressions. *In vivo* results showed in that a wide spectrum of ATG mRNA levels were up regulated in mice hippocampus exposed to cadmium, when compared to vehicle exposure (control). They are ATG3 (Figure 1a), ATG4B (Figure 1b), ATG5 (Figure 1c), ATG7 (Figure 1d), ATG9A (Figure 1e), ATG9B (Figure 1f), ATG13 (Figure 1g), ATG14 (Figure 1h), ATG16-2 (Figure 1i) and beclin1 (Figure 1j). This finding suggests that cadmium exposure activated hippocampal autophagy. To know whether LYC has potential in antagonizing Cd-induced autophagy, Cd-exposed mice were continuously treated with LYC. LYC treatment in vehicle-exposed mice has no effect in ATG mRNA expressions, when compared to control. However, LYC treatment did abrogate Cd-induced upregulation in ATG gene expressions, suggesting that LYC abrogates Cd-induced hippocampal autophagy activation.

Previous reports demonstrated that Akt1 [30], MAPK1 [31], protein kinase AMP-activated catalytic subunits (PRKAs) [32] regulates autophagy in different cells. Our data indicated that Cd exposure in mice hippocampus significantly increased the mRNA levels of Akt1 (Figure 1k), MAPK1 (Figure 1l), PRKAA2 (Figure 1m), PRKAB1 (Figure 1n) and PRKAG2 (Figure 1o). However, these upregulations were all decreased by LYC treatment to near control level. The mRNA levels of autophagy-related gene and gene regulating autophagy in mice hippocampus upon exposure and or treatment were confirmed by heatmap analysis (Figure 1p).

**Figure 2:**
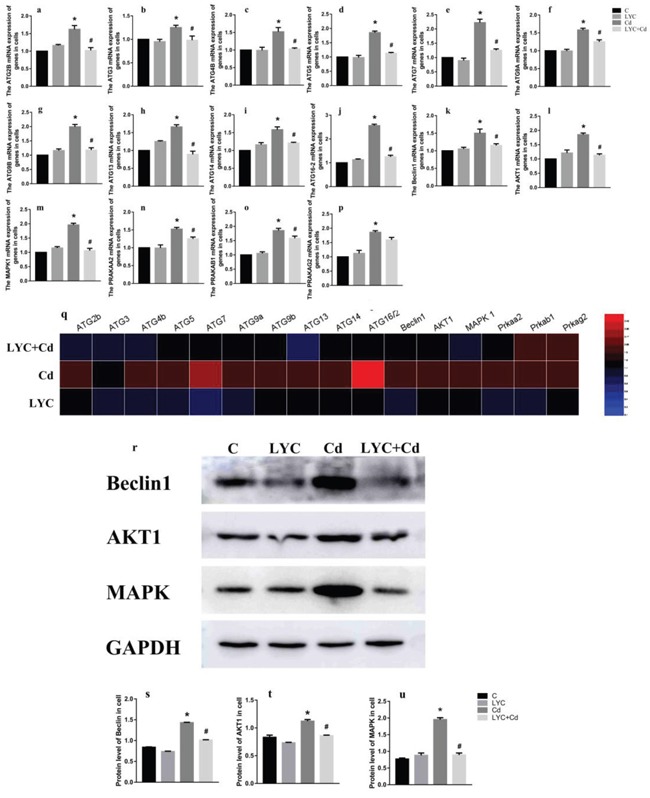
LYC abrogates cadmium exposure-induced autophagy activation in TH22 **(a-j)** ATG mRNAs, **(k)** Beclin1, **(l)** Akt1, **(m)** MAPK1, **(n)** PRKAA2, **(o)** PRKAB1, **(p)** PRKAG2, **(q)** heatmap analysis, **(r-u)** protein expression levels of beclin1, Akt1 and MAPK1. Bars represent mean ± S.D. (n=3). * represent LYC and Cd group compared to C group (P < 0.05); # represent LYC+Cd group compared to Cd group (P < 0.05).

To find the *in vitro* relevance regarding hippocampal autophagy, hippocampal neuronal cell line TH22 was utilized. TH22 cells were exposed to Cd and or treated with LYC. Similar to the *in vivo* findings, the same ATG mRNAs (Figure 2a–2k) and gene up-regulating autophagy () were all increased upon Cd exposure, which was all inhibited following LYC treatment. The only difference between *in vivo* and *in vitro* study is LYC inhibited Cd-induced ATG2B mRNA upregulation in TH22 cells (Figure 2a), but not in the mice hippocampus (data not shown). The *in vitro* study of heatmap analysis showed similar results to the *in vivo* heatmap analysis (Figure 2q). The effects of Cd and or LYC on mRNA expression levels of beclin1, Akt1 and MAPK1 were consistent with the modulation of Cd and or LYC on protein expression levels of beclin1, Akt1 and MAPK1 (Figure 2r–2u). Taken together, the above sets of data clearly showed that Cd exposure-induced hippocampal autophagy could be deactivated by LYC treatment.

### LYC inhibits cadmium exposure-induced dysfunction in hippocampal Ca^2+^ homeostasis

**Figure 3:**
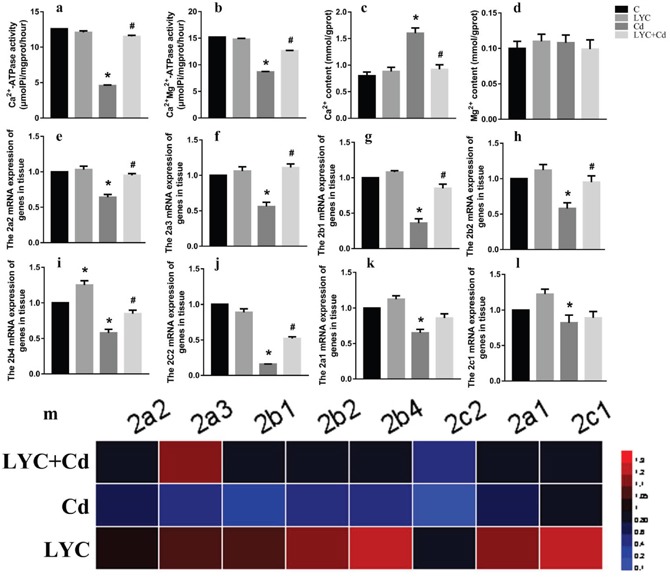
LYC inhibits Cd-induced dysfunction in hippocampal Ca2+ homeostasis **(a)** Ca^2+^-ATPase, **(b)** Ca^2+^-Mg^2+^-ATPase, **(c)** Ca^2+^ content intracellular, **(d)** Mg^2+^ content intracellular, **(e-l)** ATG mRNAs, **(m)** heatmap analysis. Bars represent mean ± S.D. (n=3). * represent LYC and Cd group compared to C group (P < 0.05); # represent LYC+Cd group compared to Cd group (P < 0.05).

Cd-induced toxicity requires the disruption of intracellular Ca^2+^ homeostasis, which is achieved by compromising Ca^2+^-ATPase activity and inducing Ca^2+^ release out of endoplasmic reticulum [3]. Both Ca^2+^-ATPase and Ca^2+^-Mg^2+^-ATPase are responsive for maintaining intracellular Ca^2+^ at low concentration for proper Ca^2+^ signaling at basal situations [33, 34]. In this regard, we sought to determine if Cd exposure modulates Ca^2+^ homeostasis in hippocampus. Results in the mice model showed that Cd exposure decreased the activities of both Ca^2+^-ATPase (Figure 3a) and Ca^2+^-Mg^2+^-ATPase (Figure 3b) in mice hippocampus tissue, when comparing to vehicle exposure. Accordingly, the intracellular Ca^2+^ content was increased by the exposure in the hippocampus tissue (Figure 3c). On the contrary, LYC treatment blocked Cd effects by increasing the Ca^2+^-ATPase and Ca^2+^-Mg^2+^-ATPase activities and decreasing intracellular Ca^2+^ concentration to control levels. Cd and or LYC has no effect on hippocampal Mg^2+^ flux (Figure 3d).

Since Ca^2+^-ATPase activity was modulated by Cd and or LYC, it was worthy investigating whether Ca^2+^-ATPase expression was also regulated accordingly. For this rationale, Ca^2+^-ATPase isoform mRNAs were determined. Cd exposure in mice hippocampus decreased isoform mRNAs as such: ATP2a2 (Figure 3e), ATP2a3 (Figure 3f), ATP2b1 (Figure 3g), ATP2b2 (Figure 3h), ATP2b4 (Figure 3i), ATP2c2 (Figure 3j). These decreased mRNAs upon Cd exposure were significantly up-regulated when treated with LYC. Besides, Cd exposure also decreased mRNAs of isoform ATP2a1 (Figure 3k) and ATP2c1 (Figure 3l), which however was not regulated with LYC treatment. The isoform ATP2b3 mRNA was not affected by Cd and or LYC (data not shown). The modulation of Ca^2+^-ATPase isoform mRNA expressions by Cd exposure and or LYC treatment was confirmed with heatmap analysis (Figure 3m).

**Figure 4:**
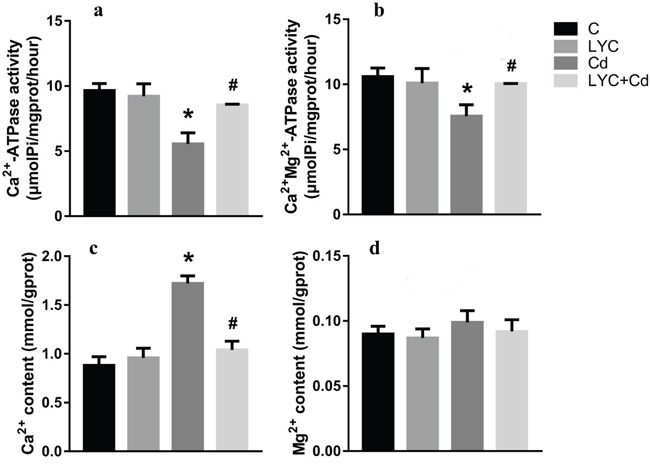
LYC inhibits Cd-induced dysfunction in Ca2+ homeostasis in TH22 **(a)** Ca^2+^-ATPase, **(b)** Ca^2+^-Mg^2+^-ATPase, **(c)** Ca^2+^ content, **(d)** Mg^2+^ content. Bars represent mean ± S.D. (n=3). * represent LYC and Cd group compared to C group (P < 0.05); # represent LYC+Cd group compared to Cd group (P < 0.05).

The *in vivo* findings of hippocampal Ca^2+^ homeostasis was recapitulated *in vitro* by using TH22 cells. Similarly, the activities of both Ca^2+^-ATPase (Figure 4a) and Ca^2+^-Mg^2+^-ATPase (Figure 4b) were down-regulated by Cd exposure, while maintained at control levels with LYC treatment. Accordingly, the intracellular Ca^2+^ concentration was upregulated by exposure, while maintained at control level with treatment (Figure 4c). Moreover, Mg^2+^ flux was not affected by either exposure or treatment (Figure 4d). In all, the above data demonstrated the potential of LYC on inhibiting Cd exposure-induced intracellular Ca^2+^ influx.

### LYC blocks cadmium exposure-triggered redox stress in hippocampus

**Figure 5:**
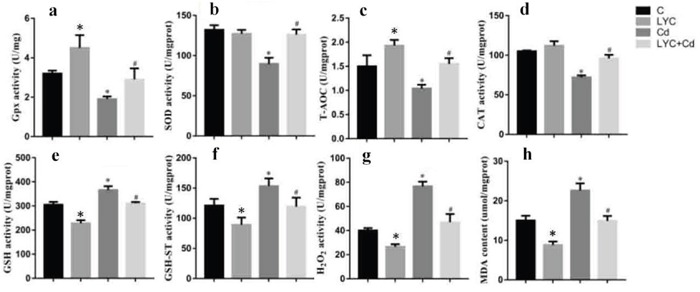
LYC blocks Cd-triggered redox enhancement in hippocampus **(a)** GSH-Px, **(b)** SOD, **(c)** T-AOC, **(d)** CAT, **(e)** GSH, **(f)** GSH-ST, **(g)** H_2_O_2_, **(h)** MDA. Bars represent mean ± S.D. (n=3). * represent LYC and Cd group compared to C group (P < 0.05); # represent LYC+Cd group compared to Cd group (P < 0.05).

Previous studies reported that the neurotoxin Cd induced-oxidative stress in mice brain or rat hippocampus contributes to neurological disorders [35, 36]. This drove our interest in investigating whether Cd triggers redox stress in mice hippocampus, and whether the antioxidant LYC has impact on the redox regulation. To unveil this, relevant antioxidant enzymes and oxidative products were quantitated in the mice hippocampus. The activities of antioxidant enzymes, GSH-Px (Figure 5a), SOD (Figure 5b), T-AOC (Figure 5c) and CAT (Figure 5d) were all downregulated in Cd-exposed hippocampus, when compared to vehicle exposure; however, LYC treatment prevented hippocampus from Cd-induced oxidative damage. Consistent to the Cd-induced oxidative stress, the levels of oxidative products in Cd-exposed hippocampus were significantly higher than that in vehicle-exposed hippocampus. These products were GSH (Figure 5e), GSH-ST (Figure 5f), H_2_O_2_ (Figure 5g) and MDA (Figure 5h). On the contrary, treatment with LYC abrogated the augment of oxidative products induced by the exposure.

**Figure 6:**
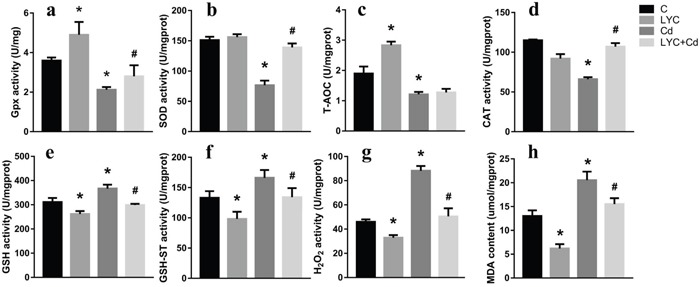
LYC blocks Cd-triggered redox enhancement in TH22 **(a)** GSH-Px, **(b)** SOD, **(c)** T-AOC, **(d)** CAT, **(e)** GSH, **(f)** GSH-ST, **(g)** H_2_O_2_, **(h)** MDA. Bars represent mean ± S.D. (n=3). * represent LYC and Cd group compared to C group (P < 0.05); # represent LYC+Cd group compared to Cd group (P < 0.05).

To see the relevance *in vitro*, oxidative stress was assessed in TH22 cells. Similarly, the activities of GSH-Px (Figure 6a), SOD (Figure 6b), T-AOC (Figure 6c) and CAT (Figure 6d) in Cd-exposed cells were significantly lower than that in control cells; however, these activities except T-AOC, in Cd-exposed cells, were modulated by LYC treatment to the control levels. On the opposite, the oxidative products, GSH (Figure 6e), GSH-ST (Figure 6f), H_2_O_2_ (Figure 6g) and MDA (Figure 6h) were significantly higher in Cd-exposed cells, when comparing to that in control cells. Moreover, these products in Cd-exposed cells were downregulated by LYC treatment. In all, the above data demonstrated the ability of LYC on blocking Cd exposure-induced oxidative stress.

### LYC treatment prevents hippocampal cells from cadmium exposure-triggered neurotoxicity

**Figure 7:**
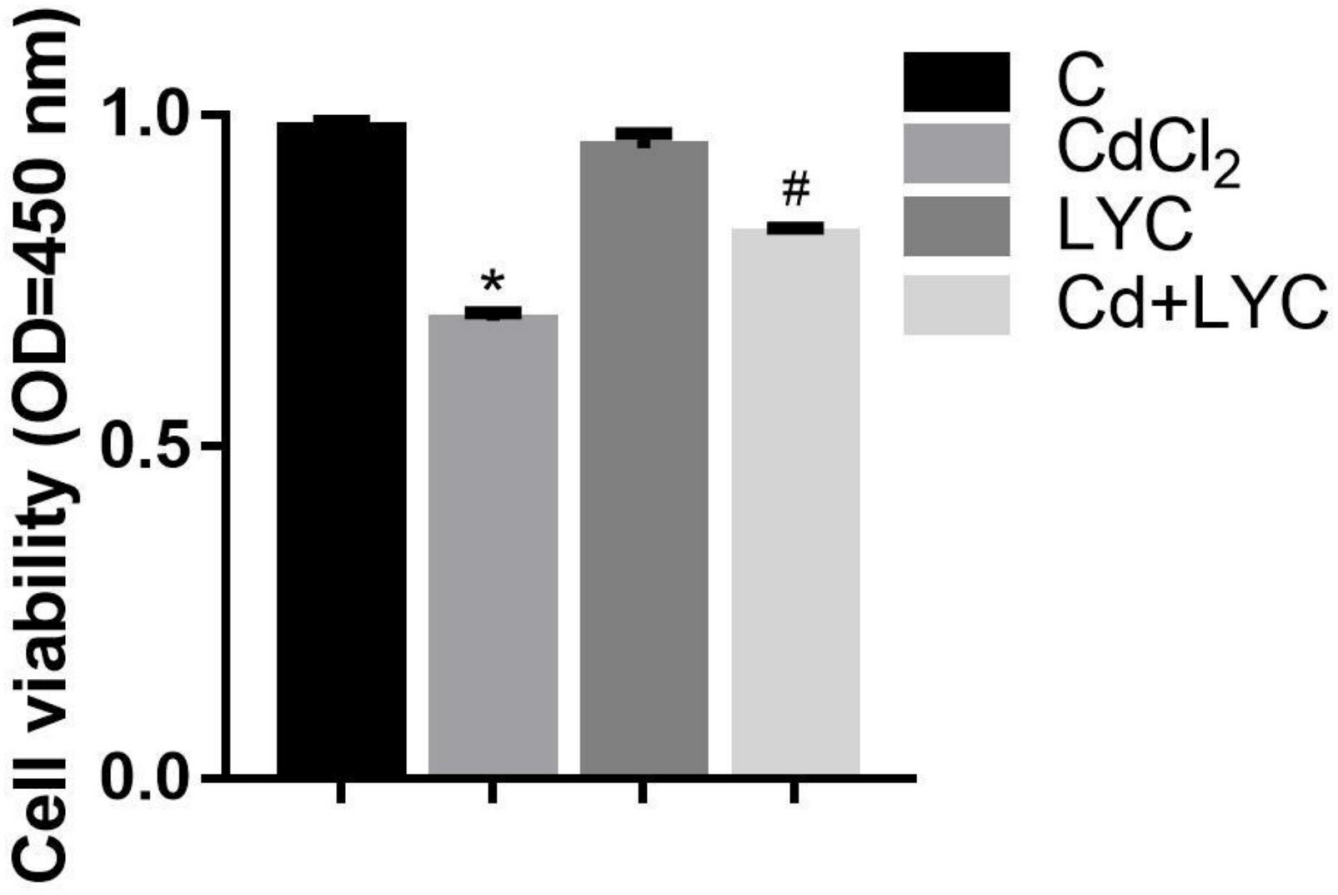
Cell viability in TH22 Bars represent mean ± S.D. (n=3). * represent LYC and Cd group compared to C group (P < 0.05); # represent LYC+Cd group compared to Cd group (P < 0.05).

Since it has demonstrated in the study that LYC treatment abrogated Cd-induced hippocampal dysfunctions in autophagy, intracellular Ca^2+^ homeostasis and redox, a key concern is whether LYC could prevent Cd-induced neurotoxicity in hippocampus. To unveil this, TH22 cells were exposed to Cd and or treated with LYC, followed by quantitating cellular ATP to assess the cytotoxicity. Results in Figure 7 showed that Cd exposure significantly decreased ATP level, when comparing to vehicle exposure group. Promisingly, the treatment of LYC significantly increased the ATP level in Cd-exposed hippocampal cells.

## DISCUSSION

A large body of studies demonstrated the toxic effects of the environmental pollutant Cd on most of human and or animal organs, such as lung, kidney, heart and brain. Autophagy activation, ion homeostasis and oxidative stress have been linked to the organ toxicity triggered by Cd exposure. In this study, we demonstrated the neurotoxicity of Cd exposure in hippocampal cells TH22. Coupling to the neurotoxicity, we also detected the dysfunctional changes in autophagy activation, intracellular Ca^2+^ concentration increase and redox augment in both mice hippocampus and TH22 cells. Dietary strategies such as intake of antioxidants have been developed in order to cure or prevent Cd exposure-induced organ toxicity. The combination of curcumin and vitamin C retains stronger antioxidant capacity than either one alone and better protects rats from Cd-induced hepatotoxicity [37]. Comparing to a single antioxidant treatment, a selected probiotic, *Lactobacillus plantarum* CCFM8610 has a better potential in chelating Cd, resulting in protective effects against acute Cd toxicity in liver and kidney [38]. There are also increasing interests in exploring promising antioxidant dietary strategies against Cd-induced neurotoxicity in brain tissue including hippocampus, such as melatonin, taurine and L-theanine [35, 39, 40]. However, none of these antioxidants has yet been applied in clinical treatment against Cd-induced organ toxicity. This calls for urgent need to explore alternative dietary antioxidant for the therapeutic or prophylactic treatment against Cd-induced neurotoxicity. Based on this, our study investigated the feasibility of LYC, and found that LYC dietary treatment at low concentration strongly inhibited Cd-induced neurotoxicity in mice hippocampal cells.

Autophagy has been determined necessary for Cd-induced hepatotoxicity, testicular injury and toxicity in mesenchymal stem cells [41–43]. There is also mounting evidence implicating autophagic dysfunction in the pathogenesis of several major neurodegenerative disorders, such as Parkinson's disease, Alzheimer's disease and Huntington's disease [3]. Besides, autophagy is found to involve in essential mental exposure-induced neurotoxicity, including lead, copper, methylmercury and cadmium [39]. Our study found that Cd exposure in both mice hippocampus and relevant HT22 cell line activated autophagy, as autophagy-related genes ATG3, ATG4B, ATG5, ATG7, ATG9, ATG13, ATG14, ATG16-2 and beclin1 were upregulated; and autophagy-regulating genes Akt1, MAPK1 as well as some 5'-AMP-activated protein kinase catalytic subunits were also upregulated. The Cd-induced overexpression of ATGs is consistent with previous study [3]. These upregulated ATGs were essential for autophagic initiation, autophagosome formation and occurrence of autophagy (PMDI: 24566140). As stated earlier, several ATGs’ expressional changes are linked to cell or organ toxicity. Interestingly, Cd exposure decreased the expression levels of ATG2B, ATG4A, ATG4C, ATG10 and ATG16-1 (data not shown), indicating that these ATGs were not essential for Cd-induced autophagy in mice hippocampus. LYC is demonstrated to be an anti-inflammatory, anti-autophagic, and anti-apoptotic agent in an experimental model of CIN [44]. However, the effect of LYC on Cd-induced autophagy is not known. Thus, our study is the first to demonstrate that LYC dietary treatment inhibited autophagy in Cd-exposed mice hippocampus. Besides inducing hippocampal autophagy, recent study demonstrated that Cd also induces apoptosis in hippocampus. This suggests that these two types of programed cell death triggered by Cd exposure co-exist in the hippocampus, and could contribute to the hippocampal homeostasis change and subsequent neurotoxicity. Actually, the co-existence of autophagy and apoptosis occurs in various cells and or situations, such as Cd-exposed primary rat osteoblasts [45] and virus-infected BSR cells [17]. Further studies demonstrated that beclin1 is a critical actor in regulating the crosstalk balance between autophagy and apoptosis [46, 47].

Ca^2+^, the crucial secondary messenger, plays an important role in cell homeostasis, signal transduction, enzyme activities, and mitochondrial dysfunctions [48]. The functioning of Ca^2+^-ATPase is important for the maintaining the concentration of intracellular Ca^2+^. Meanwhile, Ca^2+^ signaling plays a vital role in regulating apoptosis and autophagy [49]. Ca^2+^-ATPase activity can be significantly reduced when treated with oxidants, while increased by adding selenium, a kind of antioxidant [50]. One study demonstrated that Cd interacts with Ca^2+^-dependent enzymes [51]. Yuan *et al*. reported that Ca^2+^-ATPase activity was inhibited by a concentration-dependent increase of excitant Cd (reference). In our study, we demonstrated that Cd exposure decreased hippocampal activities of Ca^2+^-ATPase and Ca^2+^Mg^2+^-ATPase while increased intracellular Ca^2+^ concentration accordingly. LYC can protect against ionic homeostasis disturbance by modulating ion-transporting ATPases in hepatic and heart [52]. LYC can also regulate the activity of Ca^2+^-ATPase as well as the mRNA levels of Ca^2+^-ATPase subunits [53]. We showed that LYC treatment abrogated the dysfunctional regulation of Cd exposure on hippocampal Ca^2+^-ATPase and Ca^2+^Mg^2+^-ATPase activities as well as subunit gene expressions, and Ca^2+^ concentration.

Antioxidant enzymes and other redox regulators can act as the first line of defense to remove free radical damage. These important enzymes include GSH-Px, SOD, T-AOC and CAT [54]. Cadmium has a strong affinity for thiol groups (eg, GSH). In addition, Cd can consume thiol groups, increased the production of reactive oxygen species and hydrogen peroxide, induced oxidative stress injury [55]. Hatcher et al. suggest that elevated levels of GSH are cellular adaptation to Cd, and that Cd can enhance intracellular GSH synthesis [56]. The main role of SOD is catalyzing free radicals into hydrogen peroxide and oxygen. hydrogen peroxide can be removed by CAT to protect the cells. Nemmiche and Nzengue reported that Cd could lead to a decrease in MDA and SOD activity [57, 58]. Cd induces organ toxicity and relevant injury through creating and or augmenting oxidative stress [59, 60]. In this study, Cd exposure reduced the antioxidant enzymes (GSH-Px, SOD, T-AOC and CAT) and accordingly enhanced the production of oxidants (GSH, GSH-ST, H_2_O_2_ and MDA). LYC is an effective scavenger of peroxygen and superoxide radicals, can protect cells from H_2_O_2_-induced DNA damage [61]. LYC can enhance the body's antioxidant capacity by reducing the amount of superoxide radicals. LYC pretreatment can reduce doxorubicin-induced nephrotoxicity by improving the activity of antioxidant enzymes. LYC relieves oxidative stress in rats induced by mercury poisoning. In this study, LYC effectively abrogated Cd-mediated dysfunctional redox regulation to normal levels, indicating that LYC is a guardian of redox signaling. Previous studies demonstrated the roles of Ca^2+^ signaling in the regulation of mitochondrial ROS production, redox signaling and autophagic process [62, 63]. In addition, previous studies showed that Ca^2+^ concentration, autophagy, and redox regulation are linked to neurotoxicity as stated elsewhere. LYC treatment improves cell viability and reduces apoptosis as a result of the activation of the adaptive autophagic response on HR induced H9C2 myocardioblasts [64]. In the future study, we will focus on unveiling the inherent associations of Cd-induced Ca^2+^ homeostatic change, redox stress and autophagy activation to Cd-triggered hippocampal neurotoxicity; also, we will further optimize the LYC dietary treatment in order to initiate the clinical based study.

## MATERIALS AND METHODS

### Experimental animals

Three-week-old male Kunming mice (18-23 g) were acclimatized for 10 days before the start of the study at constant temperature of 22 ± 2°C, relative humidity of 50 ± 15%, a 12-h light/dark cycle. Temperature and relative humidity were monitored daily. Beginning at day 0, body weight was measured every day, and the consumption of drinking water and diets were measured every day. These mice were housed individually in polycarbonate cages, given access to both food and water ad libitum.

### Experimental design

100 mice were divided into 4 groups. C group was treated with auto claved water and corn oil for 21d. LYC group was treated with auto claved water and this group received 5 mg/kg LYC (Sigma) for 21d. Cd (Sigma) group was exposed to 10 mg/kg Cd for 21d. Cd + LYC group was exposed to 10 mg/kg Cd with the simultaneous pretreatment of 5 mg/kg LYC for 21d. The HT22 (Mouse hippocampal neuronal cell line) (ATCC: The Global Bioresource Center) were divided into 4 groups. C group was treated with DMEM for 24h. Cd group was treated with DMEM add 10^−6^ mol/L CdCl_2_ (Sigma) for 24h. LYC group was exposed to 10μM lycopene (Sigma) for 24h. Cd + LYC group was exposed to 10^−6^ mol/L CdCl_2_ with the simultaneous pretreatment of 10μM lycopene for 24h.

### Cell viability assay

Cell proliferation was detected by the Cell Counting Kit-8 (CCK-8). 24 h after treat with Cd and/or LYC, HT22 cells (1.0 × 10^3^) were plated into 96-well plates and cultured for 24 h. The absorbance was measured at a wave length of 450 nm.

### Determination of protein content

Protein determinations were made using the dye-binding method of Bradford. Bovine serum albumin (BSA) was used to construct the standard curve.

### Measurement of redox levels

MDA and H_2_O_2_ levels, T-SOD, GSH-Px, GSH, GSH-ST and CAT, and T-AOC activities were measured using diagnostic kits provided by Jiancheng Biotechnology Research Institute (Nanjing, China). Measurements were performed according to the protocol provided by the manufacturer in the Laboratory of the Science and Technology Experiment Centre, Shanghai University of Traditional Chinese Medicine.

### Determination of ionic concentration

The tissue was excised immediately on an ice-cold plate washed in physiological saline solution. Na^+^, K^+^, Ca^2+^ and Mg^2+^ concentration in hippocamus tissue and HT22 cell line were measured intracellularly with detection kits (Nanjing Jiancheng Bioengineering Institute, China C-002, C001-2, C004-2, C005).

### Assay to determine ATPase activity

The activities of Na^+^-K^+^-ATPase, Ca^2+^-ATPase, Mg^2+^-ATPase and Ca^2+^-Mg^2+^-ATPase were determined using the appropriate assay kits (Nanjing Jiancheng Bioengineering Institute, China) according to the manufacturer's instructions using 10% tissue homogenates. The activities of Na^+^-K^+^-ATPase, the Ca^2+^-ATPase and the Mg^2+^-ATPase were measured by quantifying the inorganic phosphorus (Pi) production from the conversion of ATP to ADP at 660 nm using the molybdenum blue spectrophotometric method and were expressed as μ molPi/mgprot/h. When one type of ATPase was tested, the inhibitors of other types of ATPase were added to depress the hydrolysis of phosphate radicals.

### Real-time polymerase chain reaction

Total RNA was isolated from cells using a Trizol reagent according to the manufacturer's instructions (Invitrogen, USA). The RNA concentrations were determined using the GeneQuant 1300. The reverse transcription reaction (40 μL) consisted of the following: 10 μg of total RNA, 1 μL of M-MLV reverse transcription,1μL of RNase inhibitor, 4 μL of dNTP, 2 μL of Oligo dT, 4 μL of dithiothreitol, and 8 μL of 5× buffer. The procedure of the reverse transcription was performed in accordance with the manufacturer's instructions (Invitrogen, USA). The reverse transcription products (cDNA) were then stored at -20°C for PCR. To design primers, we used the GenBank to get the mRNA sequence. β-actin was used as a housekeeping gene and an internal reference. Primers were designed using the Oligo 7.0 software and were synthesized by Invitrogen Biotechnology Co.Ltd.in Shanghai, China. Real-time quantitative reverse transcription PCR was used to detect the mRNA expression in cells by using SYBR Premix Ex Taq^™^ (Takara, China), and real-time PCR work was performed in an ABI PRISM 7500 real-time PCR system (Applied Biosystems). The program consisted of 1 cycle at 95°C for 30 s, 40 cycles at 95°C for 5 s and at 60°C for 34 s. The mRNA relative abundance for each gene was calculated according to the method of 2^−ΔΔCt^, accounting for gene specific efficiency and was normalized to the mean expression of GADPH or β-actin.

### Western blot analysis

Protein extracts were subjected to SDS-polyacrylamide gel electrophoresis under reducing conditions on 15% gels. Separated proteins were then transferred to nitrocellulose membranes using tank transfer for 1.5 h at 200 mA in Tris-glycine buffer containing 20% methanol. The membranes were blocked with 5% skim milk for 18-24 h and incubated overnight at 4°C with diluted primary antibody against Beclin1, Akt1, MAPK, ATP1a1, ATP1a3, ATP2a3 and ATP2c2 (1: 1000, Santa Cruz Biotechnology, USA), followed by a horseradish peroxidase (HRP) conjugated secondary antibody against rabbit IgG (1: 2000, Santa Cruz Biotechnology, USA). To verify equal sample loading, the membrane was incubated with a monoclonal GADPH antibody (1: 1000, Santa Cruz Biotechnology, USA), followed by an HRP-conjugated goat anti-mouse IgG (1: 1000, Santa Cruz Biotechnology, USA). The signal was detected using an enhanced chemiluminescence system (Cheml Scope5300, Clinx Science Instruments, Shanghai, China).

### Statistical analysis

GraphPad Prism 7.2 (GraphPad Software Inc., USA) and SPSS 20 were used to test the effects of the dietary Se levels on measures. Multiple mean comparisons were performed using Duncan's test. Data are presented as means ± S.D., and values were considered to be statistically significant if P < 0.05. The observed relationships among the parameters were confirmed and quantified according to Spearman's test. Ranking of genes by the degree of differential expression was analyzed with a heat map using the Heml 1.0 (http://hemi.biocuckoo.org/down.php).
